# Effect of Moderate Aerobic Training on Bone Metabolism Indices among Adult Humans

**DOI:** 10.12669/pjms.304.4624

**Published:** 2014

**Authors:** Ahmad H. Alghadir, Farag A. Aly, Sami A. Gabr

**Affiliations:** 1 Ahmad H. Alghadir, Department of Rehabilitation Sciences, College of Applied Medical Sciences, Rehabilitation Research Chair King Saud University, Riyadh, Kingdom of Saudi Arabia.; 2 Farag A. Aly, Faculty of Physical Therapy, Cairo University, Cairo, Egypt. Rehabilitation Research Chair King Saud University, Riyadh, Kingdom of Saudi Arabia.; 3 Sami A. Gabr, Department of Anatomy, Faculty of Medicine, Mansoura University, Rehabilitation Research Chair King Saud University, Riyadh, Kingdom of Saudi Arabia. Egypt.

**Keywords:** Aerobic exercise, Bone formation marker, Serum Osteocalcin, S-DPD, S-BAP, Calcium

## Abstract

***Objective: ***This study assessed the osteogenic effect (T-Score) and changes in bone markers in healthy subjects by 12-weeks of aerobic training.

***Methods: ***Total 65 healthy subjects (36 males, 29 females), their age ranged between 30 and 60 years with normal body mass index, were recruited to participate in this study and they were selected among healthy subjects who do not have any metabolic disorders and were not receiving any medication that could affect the bone turnover. Standardized physical examination and collection of serum samples were performed at base line and after 12 weeks of moderate aerobic training to measure bone formation markers (osteocalcin (OC) and bone specific alkaline Phosphatase (BAP) and bone resorption marker Deoxypyridinoline (DPD), and serum calcium. Each subject participated in exercise training program for 12 weeks, three times per week.

***Results:*** The results showed that the 12 weeks of moderate aerobic training produced a significant improvement in all bone metabolism indices including Serum bone-specific alkaline phosphatase, serum osteocalcin, serum free Calcium and bone mineral density among all subjects.

***Conclusion*** *:* Moderate intensity of aerobic training exerts significant positive effects on bone formation marker and bone density associated with a significant decrease in the rate of bone resorption that could assist in preventing or decelerating osteoporosis.

## INTRODUCTION

Bone is a specialized form of metabolically active connective tissue remodeled constantly through a coupled process of resorption and the formation of new bone. In adults, there was haemostatic equilibrium in bone resorption and formation around age 30, after which bone density starts to decline slowly.^1^

Exercise is a major determinant of bone mass; the mechanisms by which exercise leads to changes in bone metabolism are not fully understood. In particular, little is known about the changes in bone metabolism induced by various forms of systematic exercise.^2^ The benefit of mechanical loading or exercise on bone mass and strength has been identified in both human,^3^ and animals’ models.^4^ High intensity of exercise as jumping or resistive exercise has beneficial osteogenic effects on bone resorption and formation.^5^

The measurements of specific degradation products of bone matrix provide analytical data of the rate of bone metabolism.^6^ Besides radiological methods, several blood and urinary molecules identified as markers of bone metabolic activity for estimating the rates governing bone turnover. Out of them, osteocalcin and deoxypyridinoline (DPD) established and extensively released in the circulation and used as biochemical markers of bone resorption and formation respectively.^7^ Effective calcium homeostasis is essential for most of the biological processes, including bone metabolism.^8^ It was reported that serum calcium contributes significantly as biomarker to measure bone metabolism.^9^^,^^10^ Similarly, bone-specific alkaline phosphatase (BAP) levels are considered to reflect osteoblastic activity and can be used as a marker of bone formation.^11^ There is strong evidence that the hormonal and metabolic adaption of bone turnover to physical activity depends on age, gender, and the type of exercise performed.^12^^,^^13^ This study assessed the osteogenic effect (T-Score) and changes in bone markers in healthy subjects by moderate intensity of aerobic training that is suitable for most of the sedentary people especially those with osteoporosis.

## METHODS

***Subjects: ***Sixty five healthy subjects (36 males, 29 females), were recruited for this study. Their age ranged between 30 - 60 years (Table-I). None of them had a history of metabolic bone disease, and no drugs were taken that interfere with bone turnover. Weight and height of standardized measures were taken. Standardized physical examination was performed at baseline and after the program. All study participants gave informed consent prior to inclusion. This study was approved by ethical committee of Rehabilitation Research Chair of King Saud University.

***Training Procedure: ***Subjects participated in exercise program for 12 weeks, three times per week. Each individual training intensity was calculated as training heart rate based on his maximum and resting heart rate obtained from exercise test according to Karvonen's formula.^14^

During warming the subject performed stretching exercises and walking for 5 to 10 minutes. During the active phase, the subject was allowed to reach his pre-calculated training heart rate (THR) in bouts form with total time of 45 to 60 minutes performed as circuit training using treadmill, bicycle and stair master.

DEXA scans were used to measure BMD. The measurements of DEXA were represented as T score. The osteoporosis was diagnosed among participants using DEXA method according to T-score; Normal (0 to –0.99); Osteopenia (low bone density) (–1 to –2.49); Osteoporosis (≤ –2.5); Severe or established osteoporosis (≤ –2.5 with fracture).

**Table-I:** General characteristics of the subjects

	***Age (years)***	***Weight (kg)***	***Height (cm)***	***Body mass index (BMI) (kg/m*** ^2^ ***)***
Male (n = 36)	41.7 ± 9.3	71.7 ± 14.1.4	173.6 ± 3.9	23.7 ± 3.9
Female (n = 29).	44.4 ± 7.6	70.7 ± 10.4	173.2 ± 7.1	23.6 ± 3.5

***Bone Serum Markers:*** All serum samples were taken at the same time of day for all participants to determine Serum *osteocalcin* (ng/mL) that was determined using the MicroVue Osteocalcin enzyme immunoassay (QUIDEL Corporation, San Diego, CA), Serum *BAP *concentrations (U/L) were measured using the MicroVue BAP immunoenzymetric assay (Quidel Corporation, San Diego, CA).*)*, Serum concentrations of total* Deoxypyridinoline *(nmol/mmol) were measured with an ELISA (Metra DPD assay, Quidel Corp, San Diego CA, Cat. No, 8007). Serum calcium was determined by colorimetric methods with commercially available kits from Hoffman-La Roche (Switzerland) on Cobas Integra analyzer.

## RESULTS

**Fig.1:**
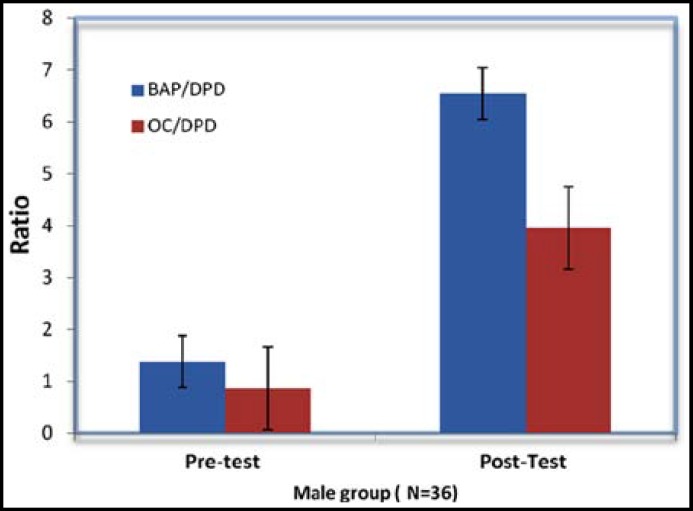
Ratio of bone-specific alkaline phosphatase (BAP) and osteocalcin (OC) to deoxypyridinoline (DPD) for male subjects (n = 36) group measured before (PRE) and after (POST) the 12-week study period. Values are mean ± SD. P < 0.05 vs Pre-test

**Fig.2:**
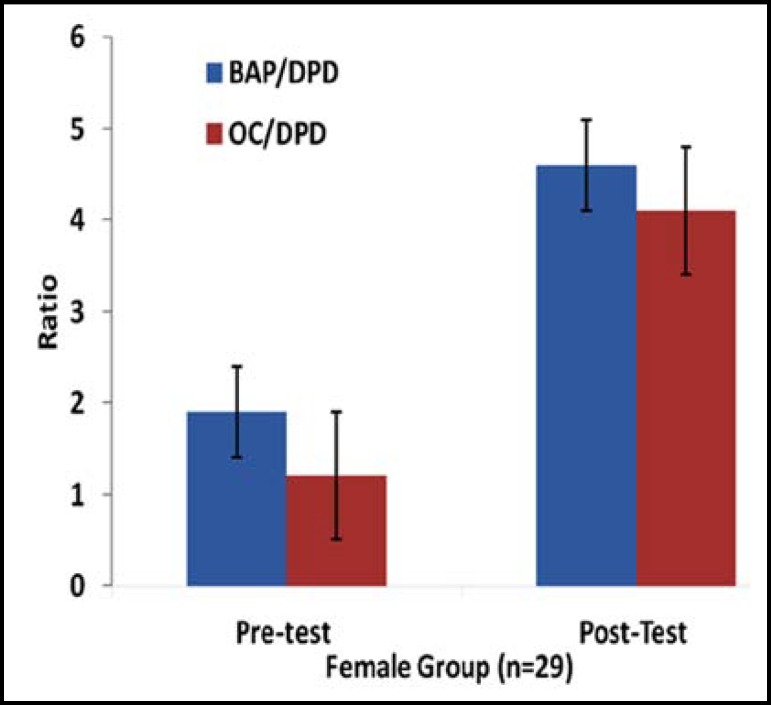
Ratio of bone-specific alkaline phosphatase (BAP) and osteocalcin (OC) to deoxypyridinoline (DPD) for female subjects (n = 29) group measured before (PRE) and after (POST) the 12-week study period. Values are mean ± SD. P < 0.05 vs Pre-test

Data were expressed as mean and standard deviation, and analyzed by using the SPSS program, version 10.0 (SPSS Inc., Chicago, IL, USA). The one-tail paired t-test was used to compare the pre- and the post-training mean values of bone metabolism indices. P value of 0.05 was used to determine the significant differences. The results showed that the 12 weeks of moderate Arabic training produced a significant improvement in all bone metabolism indices among subjects as shown in Tables II, III and Fig.1 and Fig.2.

**Table-II:** Mean, standard deviation (SD) and statistical comparison of the pre training and the post training values of the bone metabolism variables for male subjects (n = 36).

***Variables***	***Pre training value*** ***(Mean ± SD)***	***Post training value*** ***(Mean ± SD)***	***Mean difference***	***P-value***
sBAP	12.5 **±** 4.7	26.8 ± 4.1	14.3 ± 4.0	< 0.005
sDPD	9.1 **±** 3.5	6.3 ± 2	2.8 ± 1.9	< 0.005
osteocalcin	7.7 ± 1.6	24.9 ± 3.2	17.2 ± 3.5	< 0.005
S.T-Ca++	1.8 ± 0.4	2.2 ± 0.3	0.40 ± 0.2	< 0.005
S-Ca++	0.96 ± 0.3	4.9 ± 2.24	3.9 ± 2.24	< 0.005
T-Score	-1.6 ± 0.8	-1.4 ± 0.7	- 0.2 ± 0.1	<0.005
BMD ( gr/cm^2 ^)	0.68 ± 0.04	0.75 ± 0.07	0.07 ± 0.03	< 0.005

sBAP: Serum bone-specific alkaline Phosphatase (U/I); OC: serum osteocalcin (ng/ml);

sDPD: serum Deoxypyridinoline ((nmol/L); s-Ca++: serum free Calcium ( mmol/L),

sT-Ca: serum Total Calcium ( mmol/L); BMD: bone mineral density.

**Table-III:** Mean, standard deviation (SD) and statistical comparison of the pre training and the post training values of the bone metabolism variables for female subjects (n = 29).

***Variables***	***Pre training value*** ***(Mean ± SD)***	***Post training value*** ***(Mean ± SD)***	***Mean difference***	***P-value***
BAP	17.1 **±** 3.9	29.9 **±** 4.2	12.8 **±** 3.7	<0.005
DPD	9 **±** 3.1	6.5**±** 2.0	2.5 **±** 1.4	<0.005
osteocalcin	9 **±** 1.6	25.5**±** 3.0	16.5 **±** 3.4	<0.005
T.S.Ca++	1.76 **±** 0.46	2.2 0**±**.3	0.44 **±** 0.3	<0.005
S-Ca++	1.0**±** 0.3	1.3 **± **0.2	0.3 **±** 0.1	<0.005
T-Score	-1.5 **±** 0.6	-1.3 **±** 0.5	- 0.2 **±** 0.1	< 0.005
BMD ( gr/cm^2 ^)	0.82 ± 0.06	0.91 ± 0.08	0.08 ± 0.02	< 0.005

sBAP: Serum bone-specific alkaline phosphatase (U/I); OC: serum osteocalcin (ng/ml); sDpd:

serum deoxypyridinoline (nmol/L); s-Ca++: serum free Calcium (mmol/L), sT-Ca:

serum Total Calcium ( mmol/L); BMD: bone mineral density.

## Discussion

Previous cross-sectional and longitudinal^15^ studies have reported that bone remodeling is influenced by exercise. Exercise assists in formation of compensatory structures by increasing the cortical enlargement periosteally and by preventing age-related bone loss at the endosteal surfaces that depends on the frequency, duration and exercise intensity.^16^ This investigation examined the effects of moderate aerobic exercise on bone mineral density (BMD) and biochemical markers of bone turnover in healthy elderly subjects. The results indicated that BMD can be increased via moderate training even in elderly persons.

DEXA and BMD measures are reliable methods that explore the effects of osteoporosis treatment. One of the two fundamental basis of bone mineral density measurement indications is the correct estimation of fracture risk and the second is the availability of therapeutic options capable of increasing the BMD.^17^

In this study, all subjects exhibited significant improvement of T-score and BMD after moderate training. In Both groups post exercise T-score and BMD values increased approaching normal levels with a significant percentage of improvements indicating the efficacy of moderate training in enhancing BMD and osteoporosis.

Previous investigations in older adults showed that resistance exercise is an effective means for increasing BMD.^2^^,^^18^ Regular exercises leads to changes in bone turnover compatible with decreased bone resorption and increased bone formation,^4^^,^^18^^,^^19^ indicating a bone conserving effect of moderate regular exercises. This effect may be mediated through a higher activity of individual osteoblasts, while the total number of bone remodeling sites is reduced.^4^^,^^18^

Exercises have positive effects on BMD. However, there are no evidences as regard the type, severity, duration and frequency of the exercise programs.^20^ Also, it was reported that aerobics increased the BMD in femur neck in osteopenic women.^21^ Because changes in bone mass usually occur at a slow rate, measurement of BMD is inadequate to detect acute changes in bone metabolism as a result of physical activity. The skeletal response to exercise can be measured using blood markers to estimate the bone remodeling rate by comparing the resorption markers to formation markers.^22^

In this study, markers of bone formation (OC, BAP) in all subjects showed a significant increase after 12 weeks of moderate training, whereas markers of bone resorption (DPD) remained significantly lower than baseline. Post training results showed increases in formation markers, suggesting that aerobic exercise increased rate of bone remodeling. The data obtained matched with previous study that reported improvement of serum OC levels after 8-week of aerobic training.^23^ It has been also reported that exercise training led to increases in serum OC levels after periods ranging from 12–20 weeks.^24^

These observations suggest that long term aerobic exercise is associated with decelerated bone resorption and normal to elevated bone formation. Serum OC and BAP have been shown to be sensitive to alterations in bone metabolism due to physical exercise. The results of this study were also in accordance with other investigation that reported significant increases in OC and BAP following 16 wk of resistance training^2^^,^^18^, and inversely with that reported significant improvement in either muscle strength or BMD without change in bone markers.^4^^,^^25^ The change in serum OC levels may be affected not only by osteoclasts activity, the energy status of the body but also with glucose metabolism.^26^^,^^27^ Similarly, the results came in agreement with that of others who reported a significant decrease in the rate of bone resorption (DPD) and increase in bone mass with moderate exercise among old subjects.^4^^,^^5^^,^^13^

The results were also consistent with the other studies observing significant increases in the biochemical markers of bone remodeling.^28^ It has been found combined resistance and aerobic training stimulated significant increases in bone formation markers, and decrease in bone resorption marker respectively.^18^^,^^23^

This study showed that moderate training significantly increased serum calcium, that was in agreement with other studies.^29^^,^^30^ It seems that exhausting exercise sends calcium from its reserves towards blood so as to fulfill athlete's needs in strenuous and tiresome sports. Balancing and regulating calcium concentration in blood is the responsibility of parathyroid hormone (PTM). It seems that intense stress resulting from the exercise and physical activity stimulates PTM gland and increases serum calcium.^30^

In conclusion, moderate intensity of aerobic training exerts significant positive effects on bone formation marker and bone density associated with a significant decrease in the rate of bone resorption that could assist in preventing or decelerating osteoporosis.
